# Supplementary material for: A Traditional Korean Diet Alters the Expression of Circulating MicroRNAs Linked to Diabetes Mellitus in a Pilot Trial
Source: Nutrients. 2020 Aug 24;12(9):2558. doi: 10.3390/nu12092558 (PMC7551128; doi:10.3390/nu12092558)
Supplement: Supplementary file 1 [file nutrients-12-02558-s001.pdf]

Table S1. MiRs used for the validation.

miR	Accession number	Sequence
hsa_miR-25_3p	MIMAT0000081	5'CAUUGCACUUGUCUCGGUCUGA
hsa-miR-148a-3p	MIMAT0000243	5'UCAGUGCACUACAGAACUUUGU
hsa-miR-126-3p	MIMAT0000445	5'UCGUACCGUGAGUAAUAAUGCG
hsa-miR-18a-5p	MIMAT0000072	5'UAAGGUGCAUCUAGUGCAGAUAG
hsa-miR-107	MIMAT0000104	5'AGCAGCAUUGUACAGGGCUAUC
hsa-miR-26b-5p	MIMAT000008	5'UUCAAGUAAUUCAGGAUAGGU
hsa-miR-374a-5p	MIMAT0000727	5'UUAUAAUACAACCUGAUAAGUG
hsa-miR-26a-5p	MIMAT0000082	5'UUCAAGUAAUCCAGGAUAGGCU
hsa-miR-31-5p	MIMAT0000089	5'AGGCAAGAUGCUGGCAUAGCU
hsa-miR-200a-3p	MIMAT0000682	5'UAACACUGUCUGGUAACGAUGU
hsa-miR-17-3p	MIMAT0000071	5'ACUGCAGUGAAGGCACUUGUAG
hsa-miR-92a-3p	MIMAT0000092	5'UAUUGCACUUGUCCCGGCCUGU
hsa-miR-193a-5p	MIMAT0004614	5'UGGGUCUUUGCGGGCGAGAUGA
hsa-miR-19b-3p	MIMAT0000074	5'UGUGCAAAUCCAUGCAAAACUGA
